# Indoor airborne dust in veterinary facilities as a reservoir of bacterial pathogens and antimicrobial resistance

**DOI:** 10.3389/fvets.2026.1787366

**Published:** 2026-04-01

**Authors:** Hline Phyu Phyu Thant, Shadi Guillaume Kaafarani, Jules Mille, Thipruethai Phanitchat, Jiratchaya Puangseree, Si Thu Hein, Rangsiya Prathan, Songsak Srisanga, Rungtip Chuanchuen

**Affiliations:** 1 International Graduate Program of Veterinary Science and Technology, Faculty of Veterinary Science, Chulalongkorn University, Bangkok, Thailand; 2 Research Unit in Microbial Food Safety and Antimicrobial Resistance, Department of Veterinary Public Health, Faculty of Veterinary Science, Chulalongkorn University, Bangkok, Thailand; 3 National Veterinary School of Toulouse, Toulouse, France; 4 Department of Medical Entomology, Faculty of Tropical Medicine, Mahidol University, Bangkok, Thailand; 5 Department of Anatomy, University of Veterinary Science, Yezin, Nay Pyi Taw, Myanmar

**Keywords:** airborne dust, antimicrobial resistance, bacterial concentration, bacterial pathogens, veterinary facilities

## Abstract

**Introduction:**

Airborne dust in veterinary facilities can act as a reservoir for pathogens and contribute to the spread of antimicrobial resistance (AMR). This study examines airborne bacteria and their resistance profiles in veterinary hospitals and clinics.

**Methods:**

A total of 179 airborne dust samples were collected from treatment rooms (*n* = 103) and inpatient wards (*n* = 76) across 103 veterinary facilities. Total bacterial loads were quantified by direct plate counts and averaged per sample. ESKAPE pathogens, along with Escherichia coli, Salmonella, and Streptococcus, were isolated and assessed for resistance to clinically important antimicrobials, disinfectant minimum inhibitory concentrations (MICs), and plasmid conjugative transfer.

**Results:**

Airborne bacterial concentrations varied widely, ranging from 33.37 to 2,881.82 CFU/m^3^. High bacterial loads (>1,000 CFU/m^3^) were observed in a small proportion of treatment rooms (9.7%) and inpatient wards (9.2%), with mean concentrations of 1,243.8 CFU/m^3^ and 1,550.8 CFU/m^3^, respectively. *Staphylococcus* and *Enterococcus* were the most frequently detected genera (71.8% and 55.3%, respectively), while *Acinetobacter* predominated among Gram-negative bacteria (37.9%). Airborne isolates resistant to clinically important antimicrobials were isolated, including ciprofloxacin-, ceftazidime-, or colistin-resistant *Escherichia coli*; vancomycin-resistant *Enterococcus* spp.; meropenem-, imipenem-, levofloxacin-, or tigecycline-resistant *Acinetobacter* spp.; and mupirocin- or cefoxitin-resistant *Staphylococcus* spp. Notably, *Acinetobacter* isolates demonstrated horizontal transfer of ampicillin- and colistin-resistance-encoding plasmids to *E. coli*. MIC distributions for triclosan, chlorhexidine, and benzalkonium chloride were narrow, indicating no or limited reduced susceptibility to these disinfectants. Significant associations between disinfectant MICs and AMR were observed (*p* < 0.05), supporting potential co-selection and cross-resistance. In *Enterococcus*, these associations were confined to chlorhexidine and correlated with resistance to certain antibiotics. In Enterococcus, these associations were confined to chlorhexidine and correlated with resistance to certain antibiotics.

**Discussion:**

Indoor airborne dust in veterinary facilities may serve as a potential reservoir of pathogens, posing risks to animal and human health and underscoring the need for strengthened antimicrobial stewardship, infection control, ventilation, and routine AMR bioaerosol surveillance within a One Health framework.

## 1 Introduction

Antimicrobial resistance (AMR) is a critical global health challenge of the 21st century, compromising the effectiveness of antimicrobial therapies that are essential in both human and veterinary medicine (1). Evidently, the widespread and indiscriminate use of antimicrobials across human medicine and veterinary practice creates selective pressure that drives the emergence of AMR. Once established, AMR bacteria and genes can spread across human, animal and environmental interfaces, reinforcing AMR as a complex global One Health issue (2).

Antimicrobial-resistant bacteria and their resistance determinants could enter environmental compartments through multiple pathways, including effluents from healthcare settings, livestock production systems, aquacultural farming facilities, and pharmaceutical manufacturing plants, as well as through the application of manure and sewage sludge to agricultural land (3). Evidence of spread of AMR spans across a wide range of environmental matrices, such as airborne multidrug resistant (MDR) bacteria in farm settings (4), extended-spectrum β-lactamase (ESBL)-producing *Escherichia coli* in downstream surface waters (5), and the persistence of resistance genes in soils (6). Collectively, these evidences underscore the environment as a critical interface in the One Health continuum. Increasing reports demonstrate that airborne particles and bioaerosols could carry resistant bacteria, functioning as both reservoir and transmission route of AMR across wide areas. Airborne AMR bacteria were detected in diverse settings, for example, urban settings (7), livestock farms (4) and, healthcare facilities (8), highlighting their potential to spread far beyond their points of origin. Recognizing the environmental dimension of AMR, the World Health Organization (WHO) has identified AMR bacteria and their resistance genes as emerging environmental pollutants and emphasized the need for a coordinated One Health approach to curb the emergence and spread of AMR through the environmental pathways (9).

In current modern society, pets play an important role in providing companionship and emotional support, particularly to the elderly, children and individuals with special needs. One point-prevalence survey indicated that 30%−50% of hospitalized dogs and cats receive at least one antimicrobial, most frequently β-lactams and fluoroquinolones (10). The growing popularity of companion animals has intensified human–animal contact, raising concerns about cross-species transmission of AMR microorganisms. The rapid expansion of veterinary hospital services, driven by rising pet ownership and demand for animal healthcare, further increases this risk (11). Airborne dust in veterinary hospitals/clinics, where human-animal close contact frequently occur, has been shown to act as a vehicle for the spread of AMR bacteria such as methicillin-resistant *Staphylococcus aureus* (MRSA), *S. pseudintermedius* (MRSP), and vancomycin-resistant *Enterococcus* (VRE), potentially posing risks to veterinary staff, pet owners, and hospitalized animals (12, 13). This could be amplified by repeated and excessive antimicrobial use, which accelerates the selection of resistant bacterial populations (14).

Previous studies have shown that high bacterial loads in indoor environments are associated with increased risks of respiratory infections and other infectious diseases (15) as well as allergies, and other health outcomes (e.g., cancers) (16). A WHO expert-group review suggested that a total indoor microbial loads should remain below ~1,000 CFU/m3 to minimize contamination risk (17). While this is not a binding standard and does not specifically address veterinary settings, it highlights the value of sustained environmental monitoring and control of indoor bacterial concentrations including in veterinary hospitals/clinics to protect pet owners, staff and animal patients from exposure to potentially harmful microorganisms.

Reducing antibiotic use in livestock and human healthcare is a global priority in addressing AMR. Consequently, disinfectants have gained popularity for controlling microbial contamination across diverse fields. Within veterinary facilities, disinfectants such as triclosan, chlorhexidine, and benzalkonium chloride are used to maintain hygiene and limit pathogen transmission (18). However, excessive and improper use of these disinfectants may impose selective pressure favoring disinfectant-resistant bacterial strains that may develop cross-resistance to antimicrobials (19). Therefore, the implementation of evidence-based disinfection strategies is critical to mitigate the dissemination of AMR bacteria within veterinary facility environments.

Research on AMR in veterinary settings has primarily concentrated on clinical isolates from either livestock or pet animals and indoor environments such as livestock farms, schools, hospitals, and biological laboratories (4, 20, 21). Knowledge on AMR in airborne dust in veterinary hospitals/clinics is still limited. Therefore, this study aims to investigate airborne bacteria and their AMR characteristics within veterinary hospitals/clinics environment.

## 2 Materials and methods

### 2.1 Sampling location

**Figure 1:**
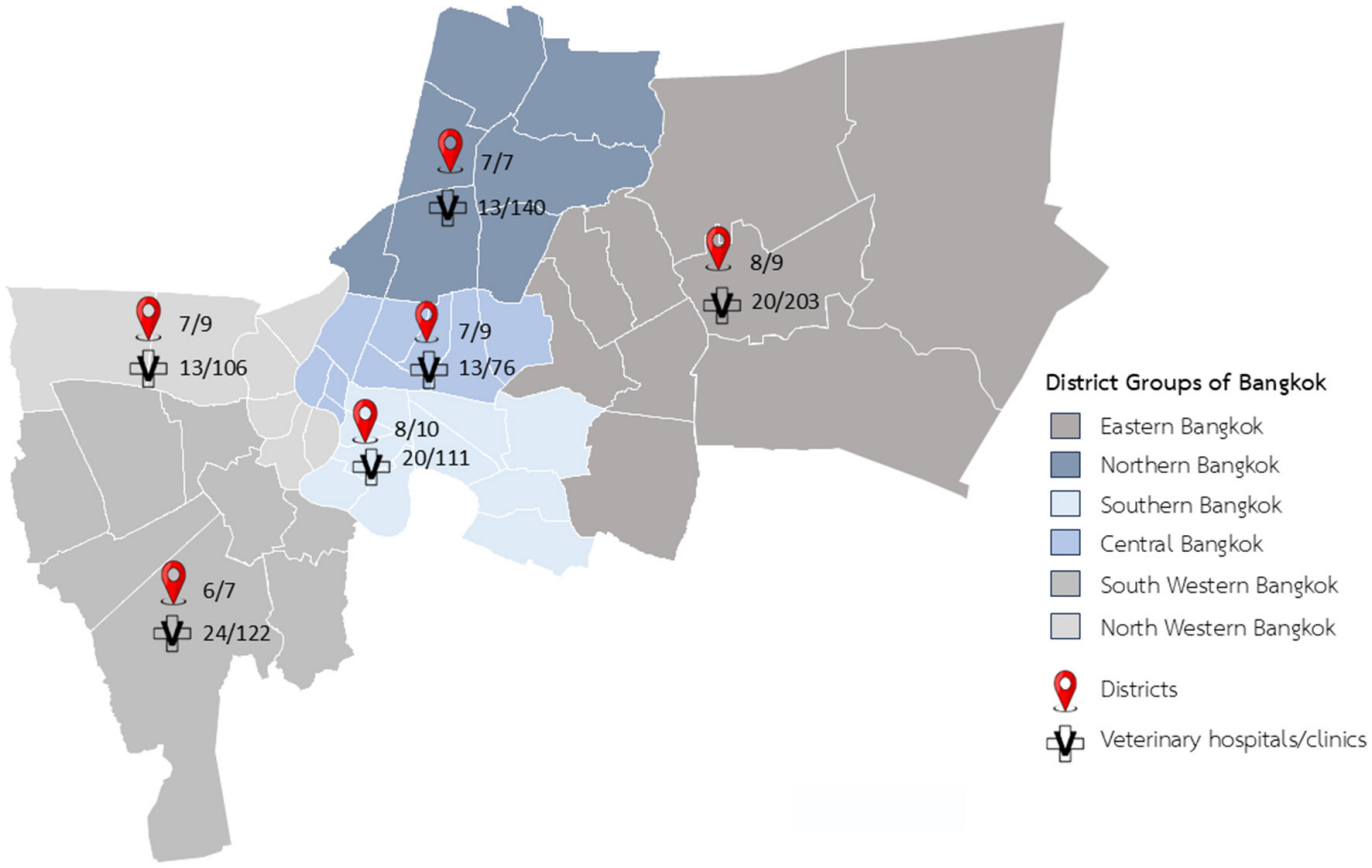
Geographical distribution of the sampled veterinary hospitals/clinics across six regions of Bangkok. 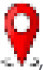, No. of sampled districts/total districts within each districts group. 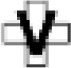, No. of sampled hospitals/total hospitals within each districts group.

In this cross-sectional study, airborne dust samples were collected at 103 veterinary hospitals/clinics within six district groups of Bangkok from October 2023 to June 2024 (Figure 1). Veterinary hospitals are defined as full-service facilities equipped with specialized rooms (such as treatment, surgery, diagnostic laboratories and inpatient wards), providing advanced medical care, surgical procedures and hospitalization. Veterinary clinics typically comprise consultation and treatment rooms for routine check-ups and minor procedures, lacking inpatient services. Of all participating veterinary hospitals/clinics (*n* = 103), 76 facilities had both treatment rooms and inpatient wards, whereas 27 had treatment rooms only. In the facilities without an inpatient ward, the airborne dust samples were obtained solely from the treatment room. A total of 179 airborne dust samples were collected, including 103 from treatment rooms and 76 from inpatient wards. Air sampling was conducted based on veterinary hospitals/clinics availability under routine management conditions. Sampling plan was initiated subsequent to telephone coordination with veterinary hospital/clinic owners or managers. Upon confirmation of participation, an official invitation letter was issued, and sample collection was scheduled. On the sample collection day, informed consent was obtained from the responsible person. Test results were shared with participating facilities upon request. This study protocol was reviewed and approved by the institutional biosafety committee (IBC), Faculty of veterinary science, Chulalongkorn university, approval number IBC 2431039.

### 2.2 Sample collection

Airborne dust samples were collected using a BioStage^®^ single-stage viable cascade impactor (SKC Inc., Eighty-Four, PA, USA) that contained 400 pores of 0.6 μm in diameter. The impactor was connected to a Quick Take 30 pump with the flow rate set to 28.3 L/min. A 100 mm × 15 mm petri dish containing tryptic soy agar (TSA, Difco™, MD, USA) was placed inside the device to facilitate airborne dust collection. During the sampling, the impactor was set approximately 1.5 m above the ground that is the respiratory height of an average person (22). Three TSA plates were used for collecting airborne dust in each room from each animal hospital/clinic. The sampling time was 6 min for each plate. Sampling plates were sealed, bagged, and transported in foam boxes to the laboratory within 6 h after collection. The environmental parameters including ambient air temperature (°C) and relative humidity (RH, %) were recorded using a Kestrel 3000 Weather Meter (Nielsen-Kellermen, PA, USA).

### 2.3 Quantification of total bacteria

The sampling plates were incubated at 37 °C for 24 h, and bacterial colonies on each plate were directly enumerated. Bacterial concentration was calculated from the average count on TSA as cometer of air, (CFU/m3), using the following formula (23).


$$\begin{array}{l} \text{Bacterial concentration }\left(\text{CFU}/\text{m}3\right)=\\ \frac{\text{No}.\text{ of colonies }\times \;1,000}{\left(\text{Flow rate }\times \text{ Sampling duration in min}\right)} \end{array}$$

Total bacterial concentration was classified into three distinct categories: low, <500 CFU/m3; moderate, 500–1,000 CFU/m3; and high, >1,000 CFU/m3 (17).

### 2.4 Isolation and identification of target bacteria

The isolation and identification of target bacterial species, including *Escherichia coli* (24), *Salmonella* (25), ESKAPE bacteria (i.e., *Enterococcus* species (26), *Staphylococcus* species (27), *Klebsiella* species (28), *Acinetobacter species* (29), *Pseudomonas* species (30) and *Enterobacter* species (28) was conducted as previously described. A loopful of bacterial colonies from each of all three TSA plates was pre-enriched in Buffered Peptone Water (BPW, Difco™) and Brain Heart Infusion broth (BHI, Difco™) at a final dilution of 1:10, incubated at 37 °C for 24 h and then, streaked onto selective media: CHROMagar™ *E. coli* (CHROMagar, Paris, France), CHROMagar™ *Salmonella* (CHROMagar, Paris, France), CHROMagar™ *Acinetobacter* (CHROMagar, Paris, France), CHROMagar™ *Pseudomonas* (CHROMagar, Paris, France), mannitol salt agar (BBL™, MD, USA) and MacConkey agar (Difco™). For *Enterococcus* spp., a loopful from BHI broth was streaked onto Slanetz & Bartley agar (OXOID^®^, Hampshire, UK). All plates were incubated at 37° C for 18–24 h. Typical colonies obtained from each selective medium, excluding *Enterococcus* spp. were subjected to species-level confirmation by using MALDI -TOF MS (Bruker Daltonics, Bremen, Germany) (31). *Enterococcus* species was identified by PCR using primer sets including *Enterococcus* species, EN-1 5′-TACTGACAAACCATTCATGATG- 3′ and EN-2 5′-AACTTCGTCACCAACGCGAAC-3′; *E. faecalis*, FL-1 5′-ACTTATGTGACTAACTTAACC-3′ and FL-2 5′-TAATGGTGAATCTTGGTTTGG-3′ and *E. faecium*, FM-1 5′-GAAAAAACAATAGAAGAATTAT-3′ and FM-2 5′-TGCTTTTTTGAATTCTTCTTTA-3′ (26). A single purified colony of each bacterial species from each positive sample was stored as 20% glycerol stock at −80° C for further analysis.

### 2.5 Antimicrobial susceptibility testing

Antimicrobial susceptibility testing (AST) was conducted using one representative isolate from each bacterial genus. For hospital/clinic-level AMR profiling, a single isolate per genus was selected from each hospital (*n* = 190). To ensure data consistency and avoid overrepresentation, the isolate selection prioritized colonies from treatment rooms. If a particular genus was not recovered from the treatment room in any hospitals/clinics, the corresponding isolate from the inpatient ward was collected. For the comparison between treatment rooms and inpatient wards, only the isolates from the 76 hospitals/clinics having both room types were included. Within each hospital/clinic, one isolate per bacterial genus was collected from each room type and included for AST. A total of 210 isolates were obtained, of which 95 were from treatment rooms and 115 were from inpatient wards.

Determination of MICs was conducted using the Sensititre™ Complete Automated AST System (Thermo Fisher Scientific, MA, USA) for all bacteria except *Enterococcus* spp. Different Sensititre™ MIC plates were used for different bacteria as follows EUVSEC2 and EUVSEC3 for *E. coli, Salmonella* and *Klebsiella* species, GNX2F for *Acinetobacter* and *Pseudomonas* species and EUST2 for *Staphylococcus* species. All antibiotic plates were purchased from Trek Diagnostic Systems, West Sussex, UK. MIC value of *Enterococcus* species was determined using two-fold agar dilution method (32).

Abbreviations of antimicrobials used in this study were as follows: amikacin, AMK; ampicillin, AMP (4–32 μg/mL); azithromycin, AZM (2–64 μg/mL); aztreonam, AZT (2–16 μg/mL); cefepime, FEP (2–16 μg/mL); cefoxitin, FOX (0.5–64 μg/mL); ceftazidime, CAZ (0.25–128 μg/mL); chloramphenicol, CHL (8–128 μg/mL); ciprofloxacin, CIP (0.015–8 μg/mL); clindamycin, CLI (0.12–4 μg/mL); colistin, COL (1–16 μg/mL); doripenem, DOR (0.12–2 μg/mL); doxycycline, DOX (2–16 μg/mL); ertapenem, ETP (0.015–2 μg/mL); erythromycin, ERY (0.25–8 μg/mL); fusidate, FUS (0.25–4 μg/mL); gentamicin; GEN (0.5–32 μg/mL), imipenem, IMI (0.12–16 μg/mL); kanamycin, KAN (4–32 μg/mL); levofloxacin, LVX (1–8 μg/mL); linezolid, LZD (1–8 μg/mL); nalidixic acid (4–128 μg/mL), NAL; meropenem, MEM (0.03–16 μg/mL); minocycline, MIN (2–16 μg/mL); mupirocin, MUP (0.5–256 μg/mL); penicillin, PEN (0.06–1 μg/mL); piperacillin/tazobactam, PT4 (8/4–64/4 μg/mL); polymycin B, POL (0.25–4 μg/mL); quinupristin/dalfopristin, SYN (0.5–4 μg/mL); rifampin, RIF (0.015–0.5 μg/mL); streptomycin, STR (4–32 μg/mL); sulfamethoxazole, SMX (64–512 μg/mL); temocillin, TEM (0.5–128 μg/mL); tetracycline, TET (2–64 μg/mL); tiamulin, TIA (0.5–4 μg/mL); ticarcillin/clavulanic acid, TCC (16/2–128/2 μg/mL); tigecycline, TGC (0.25–8 μg/mL); tobramycin, TOB (1–8 μg/mL); trimethoprim, TMP (0.25–32 μg/mL); trimethoprim/sulfamethoxazole, SXT (0.5–4 μg/mL); vancomycin, VAN (1–8 μg/mL). Interpretation was according to clinical and laboratory standards institute (CLSI) and European committee on antimicrobial susceptibility testing (EUCAST) (32, 33). *E. coli* ATCC 25922, *P. aeruginosa* ATCC 27853, *S. aureus* ATCC 29213 and *E. faecalis* ATCC 29212 served as quality control (American Type Culture Collection, Manassas, VA, USA).

Multidrug resistance (MDR) is defined as resistance to at least three different antimicrobial classes (34). A low AMR level was defined as a prevalence greater than 1% up to 10% (35).

### 2.6 Determination of MICs of disinfectants

MICs of disinfectants was determined using a twofold agar dilution method according to CLSI antimicrobial susceptibility testing procedures with some modifications (32). The disinfectants tested were as follows: triclosan (TCS, 0.0156–128 μg/mL), benzalkonium chloride (BKC, 0.5–512 μg/mL), and chlorhexidine digluconate (CHX, 0.25–2,048 μg/mL). All disinfectants were commercially obtained from Sigma-Aldrich^®^ (Saint Louis, MO, USA). *E. coli* ATCC25922, *S. aureus* ATCC29213 and *P. aeruginosa* ATCC27853 were used as control strains.

### 2.7 Test of horizontal plasmid transfer

Conjugation experiments were conducted to assess horizontal transfer of R-plasmids (36). The isolates resistant to ceftazidime, cefotaxime, colistin, meropenem, ampicillin and tetracycline, including *E. coli* (*n* = 2) and *Pseudomonas* spp. (*n* = 5) and *Acinetobacter* spp. (*n* = 17) served as donors. *Salmonella* Enteritidis SE12 rif ^r^ (RIF MIC=256 μg/mL) (37) was used as recipient for *E. coli* donors and *E. coli* MG1655rif ^r^ (RIF MIC=256 μg/mL) (38) was used as recipients for *Pseudomonas* spp. and *Acinetobacter* spp. donors. Transconjugants were selected on Luria Bertani agar containing RIF, 32 μg/mL and one of the following antibiotics: AMP, 150 μg/mL; TET, 15 μg/mL; CAZ, 1 μg/mL; CTX, 1 μg/mL; COL, 2 μg/mL; and MEM, 4 μg/mL. Transconjugants were determined for their susceptibilities to corresponding antibiotics. The conjugation efficiency was subsequently estimated.

### 2.8 Statistical analysis

Two-way ANOVA with Scheffé *post hoc* test was used to compare bacterial concentrations and environmental parameters between treatment rooms and inpatient wards. Associations between AMR, room type, and disinfectant MICs were evaluated using Fisher's exact test. Correlations among MICs of disinfectants/heavy metals and antibiotics, as well as between MICs of different antibiotics, were assessed by Spearman rank correlation. Analyses were conducted in SPSS v29.0 (IBM Corp., Armonk, NY, USA), with statistical significance set at *p* < 0.05.

## 3 Results

### 3.1 Bacterial concentration

Bacterial concentrations in indoor air varied widely across samples from veterinary hospitals/clinics, ranging from 33.37 to 2,881.82 CFU/m3 (Table 1). Most treatment rooms (*n* = 72) and inpatient wards (*n* =53) were classified into the low-concentration category, with mean bacterial loads of 238.3 CFU/m3 (95% CI: 396.91–557.81) and 246.8 CFU/m3 (95% CI: 199.63–294.03), respectively. A small subset of rooms reached the high-concentration category, 10 treatment rooms and seven inpatient wards with corresponding mean values of 1,243.8 CFU/m3 (95% CI: 1,135.15–1,352.48) and 1,550.8 CFU/m3 (95% CI: 1,420.97–1,680.72).

**Table 1:** Mean bacterial concentration and environmental parameters by different room types and concentration levels in veterinary hospitals/clinics (*n* = 179).

Level	Bacterial concentration (CFU/m3)^a^	Room type^b^	No of rooms	Bacterial concentration (CFU/m3)		*p*-value	Relative humidity (%)		*p* value	Temperature (°C)		*p* value
				Mean ±SD	95% CI		Mean ±SD	95% CI		Mean ±SD	95% CI	
Low	1–500	T × R (*n* = 103)	72	238.3 ± 111.7	557.81–396.91	0.004^*^	54.9 ± 7.2	53.05–56.67	0.048^*^	27.5 ± 2.5	26.99–28.05	0.025^*^
		IpW (*n* = 76)	53	246.8 ± 123.4	199.63–294.03		54.3 ± 8.4	52.23–56.44		28.0 ± 2.2	27.33–28.57	
Medium	>500–1,000	T × R (*n* = 103)	21	723.8 ± 149.4	648.83–798.81	0.004^*^	53.0 ± 7.5	49.61–56.30	0.048^*^	27.5 ± 2.0	26.53–28.50	0.025^*^
		IpW (*n* = 76)	16	713.3 ± 158.3	627.43–799.25		56.8 ± 7.9	52.97–60.63		28.4 ± 2.6	27.28–29.53	
High	>1,000	T × R (*n* = 103)	10	1,243.8 ± 185.3	1,135.15–1,352.48	0.004^*^	56.5 ± 10.0	51.67–61.37	0.048^*^	30.1 ± 3.5	28.64–31.48	0.025^*^
		IpW (*n* = 76)	7	1,550.8 ± 637.2	1,420.97–1,680.72		49.0 ± 4.4	43.20–54.80		27.4 ± 1.5	25.71–29.11	

^*^Statistically significant at *p* < 0.05.

^a^Statistical comparisons were performed using two-way ANOVA with Scheffé post hoc test (*n* = 179).

^b^TxR, treatment room; IpW, inpatient wards.

SD, standard deviation; CI, confidence interval.

The association between airborne bacterial concentrations and environmental parameters differed between treatment rooms and inpatient wards (*p* < 0.05; Table 1). Based on the average bacterial concentration, most treatment rooms (70%) and inpatient wards (69.7%) were classified into the low-level category. In both low and high concentration categories, inpatient wards exhibited higher mean values of the airborne bacterial concentration than treatment rooms (*p* =0.004). In contrast, at the moderate level category, the average bacterial concentration in inpatient wards (713.3 ± 158.3 CFU/m3) was lower than in treatment rooms (723.8 ± 149.4 CFU/m3; *p* = 0.004).

Bacterial concentrations were significantly associated with environmental parameters (*p* < 0.05). The association between bacterial concentration level and temperature or RH in both room types was not consistent. Treatment rooms exhibited higher bacterial concentrations in conjunction with higher temperature and RH, with mean values of 30.1 ± 3.5 °C and 56.5 ± 10.0%, respectively. In contrast, increased bacterial concentrations in inpatient wards coincided with lower temperature and RH, with mean values of 27.4 ± 1.5 °C and 49.0 ± 4.4%.

### 3.2 Bacterial species isolated from airborne dust samples

Among bacterial species isolated, Gram-positive genera dominated in the airborne dust samples collected from veterinary hospitals and clinics (*n* = 103; Table 2). *Staphylococcus* (*n* = 74) and *Enterococcus* (*n* = 57) were the most frequently detected genera in veterinary hospitals/clinics. By room type, both genera were most commonly identified in treatment rooms (*n* = 84/103 and 65/103, respectively), followed by inpatient wards (*n* = 63/76 and 54/76, respectively). Among Gram-negative bacteria, *Acinetobacte*r was most frequently detected, occurring in 39 hospitals/clinics (37.86%) followed by *Pseudomonas* (*n* =10), *E. coli* (*n* = 8), and *Klebsiella* (*n* = 2).

**Table 2:** Number of veterinary hospitals/clinics positive for target bacterial genera in airborne dust samples, by room type (*n* = 103).

Bacterial genera^c^	No. of positive hospitals/clinics	Room type^a^		
		T×R	IpW	Both rooms^b^
*Acinetobacter*	39	23	25	9
*Enterococcus*	57	43	34	20
*E. coli*	8	4	7	3
*Klebsiella*	2	–	2	-
*Pseudomonas*	10	6	7	3
*Staphylococcus*	74	59	40	25

^a^TxR, treatment room; IpW, inpatient wards.

^b^Indicates that the genus was detected in both room types within the same hospitals/clinics.

^c^One isolate was collected from each positive sample.

### 3.3 Antimicrobial resistance and resistance phenotypes

For AMR analysis at the hospital/clinic level, one isolate per genus was collected from each positive hospital/clinic (*n* = 190). The composition of bacterial species was presented in Table 3. The majority isolates were *Staphylococcus* (*n* = 74), among which *S. haemolyticus* (*n* = 49) was the most prevalent. This is followed by *Enterococcus* (*n* = 57) and *Acinetobacter* (*n* = 39) with *A. baumannii* (*n* = 19) being the predominant Gram-negative species.

**Table 3:** Bacterial species composition at hospitals/clinics level (*n* = 190) and room-level (*n* = 210) for AMR analyses.

Genus	Species	Hospital level^a^ (*n* = 103)	Room level^b^		
			TxR (*n* = 103)	IpW (*n* = 76)	Total
*Acinetobacter*	*Acinetobacter baumannii*	19	7	12	19
	*Acinetobacter bereziniae*	1	–	1	1
	*Acinetobacter courvalinii*	1	–	1	1
	*Acinetobacter cumulans*	1	–	1	1
	*Acinetobacter indicus*	1	–	–	-
	*Acinetobacter junii*	5	3	2	5
	*Acinetobacter nosocomialis*	1	–	1	1
	*Acinetobacter pittii*	4	2	4	6
	*Acinetobacter radioresistens*	1	–	2	2
	*Acinetobacter soli*	2	1	–	1
	*Acinetobacter variabilis*	3	2	1	3
*Enterococcus*	*Enterococcus facecalis*	11	6	5	11
	*Enterococcus faecium*	12	5	14	19
	*Enterococcus species*	34	17	15	32
*Escherichia*	*E. coli*	8	4	7	11
*Klebsiella*	*K. pneumonia*	2	–	2	2
*Pseudomonas*	*Pseudomonas aeruginosa*	3	1	2	3
	*Pseudomonas mendocia*	1	–	1	1
	*Pseudomonas putida*	1	1	1	2
	*Pseudomonas stutzeri*	5	3	3	6
*Staphylococcus*	*Staphylococcus aureus*	3	2	5	7
	*Staphylococcus borealis*	2	1	–	1
	*Staphylococcus coagulans*	1	–	–	-
	*Staphylococcus cohnii*	3	2	1	3
	*Staphylococcus felis*	1	–	–	-
	*Staphylococcus haemolyticus*	49	29	22	51
	*Staphylococcus hominis*	–	–	1	1
	*Staphylococcus pseudintermedius*	2	1	2	3
	*Staphylococcus schleiferi*	3	3	–	3
	*Staphylococcus simulans*	2	1	3	4
	*Staphylococcus ureilyticus*	5	2	4	6
	*Staphylococcus warnei*	2	1	2	3
	*Staphylococcus xylosus*	1	1	–	1
Total		190	95	115	210
Grand total		190	210		

–, not found.

^a^One isolate per genus was selected, prioritizing treatment rooms.

^b^One isolate per genus was selected from each room within the same hospital.

At the room level (*n* = 210), one isolate per genus was collected from each room type. The bacterial species distribution was similar to that observed at the hospital level. *Staphylococcus haemolyticus* (*n* = 29 for treatment rooms, *n* = 22 for inpatient wards) was predominant in both room settings, whereas *Enterococcus faecium* (*n* = 14) and *Acinetobacter baumannii* (*n* = 12) were isolated more frequently in inpatient wards.

At veterinary hospitals/clinics level, AMR rates varied by target bacteria (*n* = 190; Table 4). Among the *Staphylococcus* isolates (*n* = 74), resistance was most frequent to PEN (83.8%), followed by SMX (67.5%), TET (63.5%), ERY (59.5%), and TIA (50.0%), whereas resistance to VAN and LZD remained low (2.7%). *Enterococcus* isolates (*n* = 57) exhibited resistance to several antimicrobials, including ERY (31.58%), TET (29.82%), and AMP (17.54%), with lower resistance rates to CHL (12.28%) and, VAN (1.75%). A high level of STR resistance (MIC = 2,048 μg/mL) was observed (12.28%). None was resistant to GEN. *Acinetobacter* isolates (*n* = 39) showed higher resistance rates to TGC (25.64%), SXT (20.51%), and CIP (20.51%), but resistance to carbapenems such as DOR (7.69%), IMI (5.13%) and MEM (5.13%) was low. The number of *Pseudomonas* (*n* = 10), *E. coli* (*n* = 8), and *Klebsiella* (*n* = 2) isolates were too small to allow meaningful analysis. The *Pseudomonas* isolates were resistant to IMI (*n* = 4), SXT (*n* = 4) and DOX (*n* = 3). Both *Klebsiella* isolates (*n* =2) were resistant to AMP, AZM, SMX, TCC and TMP. Despite the limited number, the *E. coli* isolates obtained (*n* = 8) were resistant to colistin (*n* = 2), CIP (*n* = 1) and FOX (*n* = 1). None of the isolates were ESBL-producers.

**Table 4:** Antimicrobial resistance rate (%) of bacterial species at veterinary hospitals/clinics level (*n* = 190).

Antimicrobials	No. of resistant isolates (%)^a^					
	*Enterococcus* (*n* = 57)	*Staphylococcus* (*n* = 74)	*Klebsiella* (*n* = 2)	*Acinetobacter* (*n* = 39)	*Pseudomonas* (*n* = 10)	*E.coli* (*n* = 8)
Amikacin	–	–	–	2 (5.13)	0	–
Ampicillin	10 (17.54)	–	2 (100)	–	–	5 (62.5)
Azithromycin	–	–	2 (100)	–	–	8 (100)
Aztreonam	–	–	–	–	0	–
Cefotaxime	–	–	0	4 (10.26)	0	0
Cefoxitin	–	32 (43.24)	0	–	–	1 (12.5)
Ceftazidime	–	–	0	2 (5.13)	0	1 (12.5)
Chloramphenicol	7 (12.28)	10 (13.51)	0	–	–	3 (37.5)
Ciprofloxacin	–	22 (29.73)	1 (50)	8 (20.51)	1 (10)	1 (12.5)
Clindamycin	–	35 (47.3)	–	–	–	–
Colistin	–	–	0	3 (7.69)	0	2 (25)
Doripenem	–	–	–	3 (7.69)	0	–
Doxycycline	–	–	–	4 (10.26)	3 (30)	–
Erythromycin	18 (31.58)	44 (59.46)	–	–	–	–
Fusidate	–	24 (32.43)	–	–	–	–
Gentamicin	0	14 (18.92)	0	2 (5.13)	0	1 (12.5)
Imipenem	–	–	0	2 (5.13)	4 (40)	0
Kanamycin	–	33 (44.59)	–	–	–	–
Levofloxacin	–	–	–	4 (10.26)	0	–
Linezolid	–	2 (2.7)	–	–	–	–
Nalidixic acid	–	–	0	–	–	2 (25)
Meropenem	–	–	0	2 (5.13)	1 (10)	–
Minocycline	–	–	–	0	0	–
Mupirocin	–	22 (29.73)	–	–	–	–
Penicillin	–	62 (83.78)	–	–	–	–
Piperacillin/Tazobactam	–	–	–	1 (2.56)	1 (10)	–
Polymyxin B	–	–	–	1 (2.56)	0	–
Quinupristin/dalfopristin	–	11 (14.86)	–	–	–	–
Rifampin	–	0	–	–	–	–
Streptomycin	7 (12.28)	7 (9.46)	–	–	–	–
Sulfamethoxazole	–	50 (67.57)	2 (100)	–	–	5 (62.5)
Temocillin	–	–	–	–	–	1 (12.5)
Tetracycline	17 (29.82)	47 (63.51)	0	–	–	5 (62.5)
Tiamulin	–	37 (50)	–	–	–	–
Ticarcillin/Clavulanic acid	–	–	2 (100)	3 (7.69)	1 (10)	–
Tigecycline	–	–	–	10 (25.64)	–	0
Tobramycin	–	–	–	2 (5.13)	0	–
Trimethoprim	–	24 (32.43)	2 (100)	–	–	–
Trimethoprim/ Sulfamethoxazole	–	–	–	8 (20.51)	4 (40)	3 (37.5)
Vancomycin	1 (1)	2 (2.7)	–	–	–	–
MDR	9 (15.78)	65 (87.83)	2 (100)	7 (17.95)	0	8 (100)

–, not tested.

^a^All isolates were collected from treatment rooms or, if unavailable, from the inpatient ward of each positive hospital (*n* = 190).

MDR was predominantly detected in *Staphylococcus*. (87.83%). Lower proportions were observed in *Enterococcus*. (9/57, 15.78%) and *Acinetobacter* (7/39, 17.95%). All *E. coli* (8/8) and *Klebsiella* (2/2) were MDR isolates, whereas no MDR phenotype was observed among *Pseudomonas* isolates.

When comparing between treatment rooms and inpatient wards, *Staphylococcus* isolates show high resistance to PEN (35/43, 81.4% and 33/40, 82.5%) and SMX (28/43, 65.11% and 29/40, 72.5%) with minor differences between the two room types. Among *Enterococcus*, resistance to GEN and VAN was each detected in a single isolate obtained from an inpatient ward (Table 5). Among *Acinetobacter* isolates from treatment rooms, resistance to last-resort antimicrobials was observed for COL (2/15, 13.3%) and TGC (1/15, 6.7%). For *Acinetobacter* isolates from inpatient wards, no COL-resistant isolates were detected; however, resistance to TGC (12/25, 48.0%), FOX (1/25, 4%), CAZ (1/25, 4%), IMI (2/25, 8%), MEM (1/25, 4%), and LVX (6/25, 24%) was identified. Although the number of *Pseudomonas* isolates was limited, IMI resistance was observed in both settings, with 2/5 (40.0%) resistance in treatment rooms and 3/8 (37.5%) in inpatient wards. Among *E. coli* isolates, resistance to CAZ was detected in treatment rooms (1/4, 25.0%), whereas COL resistance was identified in inpatient wards (1/7, 14.3%).

**Table 5:** Comparison of antimicrobial resistance rate (%) of bacterial species between treatment rooms and inpatient wards in veterinary hospitals/clinics (*n* = 210).

Antimicrobials	No. of resistant isolates (%)^a, b^											
	*Enterococcus*		*Staphylococcus*		*Klebsiella*		*Acinetobacter*		*Pseudomonas*		*E. coli*	
	T×R (*n* = 28)	IpW (*n* = 34)	T×R (*n* = 43)	IpW (*n* = 40)	T×R (*n* = 0)	IpW (*n* = 2)	T×R (*n* = 15)	IpW (*n* = 25)	T×R (*n* = 5)	IpW (*n* = 8)	T×R (*n* = 4)	IpW (*n* = 7)
Amikacin	–	–	–	–	–	1 (100)	0	4 (16)	0	0	2 (50)	4 (57.14)
Ampicillin	6 (21.43)	8 (23.52)	–	–	–	–	–	–	–	–	4 (100)	7 (100)
Azithromycin	–	–	–	–	–	1 (100)	–	–	0	0	–	–
Aztreonam	–	–	–	–	–	–	–	–	0	0	–	–
Cefotaxime	–	–	–	–	–	0	1 (6.67)	1 (4)	0	0	0	0
Cefoxitin	–	–	20 (46.51)	18 (45)	–	0	–	–	–	–	0	1 (14.28)
Ceftazidime	–	–	–	–	–	0	1 (6.67)	1 (4)	0	0	1 (25)	0
Chloramphenicol	4 (14.29)	3 (8.8)	7 (16.28)	8 (20)	–	0	–	–	–	–	1 (25)	2 (28.57)
Ciprofloxacin	–	–	13 (30.23)	12 (30)	–	1 (50)	1 (6.67)	2 (8)	0	1 (12.5)	0	1 (14.28)
Clindamycin	–	–	18 (41.86)	22 (55)	–	–	–	–	–	–	–	–
Colistin	–	–	–	–	–	0	2 (13.33)	0	0	0	0	1 (14.28)
Doripenem	–	–	–	–	–	–	2 (13.33)	2 (8)	0	0	–	–
Doxycycline	–	–	–	–	–	–	0	6 (24)	1 (20)	2 (25)	–	–
Erythromycin	10 (35.71)	10 (29.41)	28 (65.11)	20 (50)	–	–	–	–	–	–	–	–
Fusidate			14 (32.56)	15 (37.5)	–	–	–	–	–	–	–	–
Gentamicin	0	1 (2.9)	9 (20.93)	8 (20)	–	0	0	3 (12)	0	0	0	1 (14.28)
Imipenem	–	–	–	–	–	0	1 (6.67)	2 (8)	2 (40)	3 (37.5)	0	0
Kanamycin	–	–	19 (44.18)	17 (42.5)	–	–	–	–	–	–	–	–
Levofloxacin	–	–	–	–	–	–	0	6 (24)	0	0	–	–
Linezolid	–	–	1 (2.32)	6 (13.95)	–	–	–	–	–	–	–	–
Nalidixic acid	–	–	–	–	–	1 (50)	–	–	–	–	1 (25)	1 (14.28)
Meropenem	–	–	–	–	–	0	1 (6.67)	1 (4)	1 (20)	0	–	–
Minocycline	–	–	–	–	–	–	0	1 (4)	0	0	–	–
Mupirocin	–	–	12 (27.91)	19 (47.50)	–	–	–	–	–	–	–	–
Penicillin	–	–	35 (81.40)	33 (82.5)	–	–	–	–	–	–	–	–
Piperacillin/ Tazobactam	–	–	–	–	–	–	0	3 (12)	1 (20)	0	–	–
Polymyxin B	–	–	–	–	–	–	1 (6.67)	0	0	0	–	–
Quinupristin/ dalfopristin	–	–	6 (13.95)	10 (25)	–	–	–	–	–	–	–	–
Rifampin	–	–	0	0	–	–	–	–	–	–	–	–
Streptomycin	3 (10.71)	2 (5.88)	4 (9.30)	6 (13.95)	–	–	–	–	–	–	–	–
Sulfamethoxazole	–	–	28 (65.11)	29 (72.5)	–	2 (100)	–	–	–	–	4 (100)	4 (57.14)
Temocillin	–	–	–	–	–	–	–	–	–	–	1 (25)	0
Tetracycline	8 (28.57)	10 (29.41)	28 (65.11)	21 (52.5)	–	0	–	–	–	–	2 (50)	4 (57.14)
Tiamulin	–	–	21 (48.84)	23 (57.5)	–	–	–	–	–	–	–	–
Ticarcillin/ Clavulanic acid	–	–	–	–	–	–	0	4 (16)	1 (20)	1 (12.5)	–	–
Tigecycline	–	–	–	–	–	0	1 (6.67)^c^	12 (48)^c^	–	–	0	1 (14.28)
Tobramycin	–	–	–	–	–	–	0	3 (12)	0	0	–	–
Trimethoprim	–	–	14 (32.56)	13 (32.5)	–	0	–	–	–	–	1 (25)	3 (42.85)
Trimethoprim/ Sulfamethoxazole	–	–	–	–	–	–	1 (6.67)	9 (75)	2 (40)	3 (37.5)	–	–
Vancomycin	0	1 (2.9)	1 (2.32)	3 (7.5)	–	–	–	–	–	–	–	–

–, not tested.

^a^TxR, treatment room; IpW, inpatient wards.

^b^All isolates were collected from treatment rooms and inpatient wards within the same hospitals (*n* = 76).

^c^Significantly different (*p* < 0.05).

Bold number, high-level aminoglycoside resistance in Enterococcus (MIC = 2,048 μg/mL and >1,024μg/mL for streptomycin and gentamicin, respectively).

Statistical association between AMR phenotype and different room types were examined for *Staphylococcus* spp. and *Enterococcus* spp.; however, no significant associations were observed. AMR rates were not associated with room types. Among *Acinetobacter* spp., only TGC resistance showed a significant association with inpatient wards (*p* < 0.05).

### 3.4 Correlation analysis of antimicrobial MIC values

**Figure 2:**
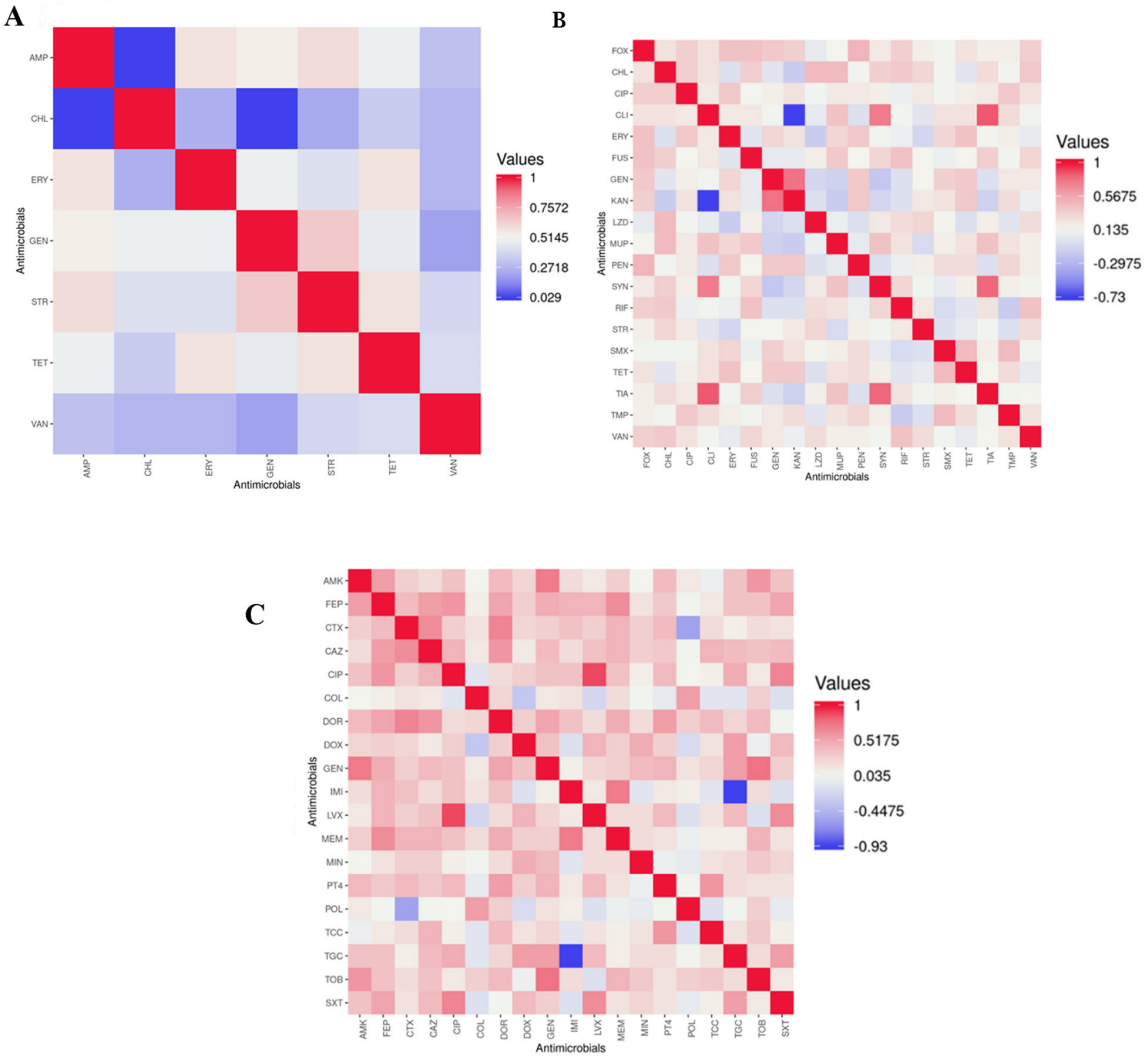
Correlation between MICs value of antimicrobials *Enterococcus* (*n* = 57) **(A)**, *Staphylococcus* (*n* = 74) **(B)**, and Acinetobacter (*n* = 39) **(C)**. Correlation heatmaps visually display the Spearman rank correlation coefficients between each pair of parameters. The scale bar on the right indicates correlation values, where 1.0 denotes strong positive correlation, −1.0 indicates strong negative correlation, and 0 represents no correlation. AMK, amikacin; AMP, ampicillin; CAZ, ceftazidime; CHL, chloramphenicol; CIP, ciprofloxacin; CLI, clindamycin; COL, colistin; CTX, cefotaxime; DOR, doripenem. DOX, doxycycline; ERY, erythromycin; FEP, cefepime; FOX, cefoxitin; FUS, fusidate; GEN, gentamicin; IMI, impipenem; KAN, kanamycin; LVX, levofloxacin; LZD, linezolid; MEM, meropenem; MIN, minocycline; MUP, mupirocine; PEN, Penicillin; PT4, piperacillin/Tazobactam; POL, polymyxin B; SMX, sulfamethoxazole; STR, streptomycin; SXT, trimethoprim/sulfamethoxazole; SYN, Quinupristin/dalfopristin; TCC, ticarcillin/clavulanic acid; TET, tetracycline; TGC, tigecycline; TIA, tiamulin; TMP, trimethoprim; TOB, tobramycin; VAN, vancomycin.

Statistical correlation was analyzed among *Enterococcus, Staphylococcus*, and *Acinetobacter* isolates. Most tested antimicrobials exhibited strong positive correlations with one another. In *Enterococcus*, significant positive correlations were detected between the MIC of STR and those of TET (*r* = 0.591) and AMP (*r* = 0.609), *p* < 0.05; Figure 2A). In *Staphylococcus* isolates, a strong positive correlation was observed between the MIC of CLI and TIA (*r* = 0.832, *p* < 0.05; Figure 2B). Among *Acinetobacter*, the MIC of MEM was positively correlated to those of FEP (*r* = 0.632) and IMI (*r* = 0.726, *p* < 0.05; Figure 2C).

### 3.5 Disinfectant MICs distribution

Due to the lack of interpretive criteria, MIC distributions of disinfectants were presented for the target bacteria (*n* = 190; Table 6). For *Enterococcus*, TCS MICs ranged from ≤ 0.0156 to 8 μg/mL, with most values clustered at 8 μg/mL. Among *Staphylococcus* isolates, TCS MICs spanned from ≤ 0.0156 to 2 μg/mL, with a pronounced concentration at ≤ 0.0156 μg/mL (36/74, 48.7%). Most *Acinetobacter* had TCS MICs of ≤ 0.0156 μg/mL (11/39, 28.2%) and 0.03125 μg/mL (8/39, 20.5%). Only *Pseudomonas* exhibited TCS MIC up to 256 μg/mL.

**Table 6:** MIC values for disinfectants in target bacterial species (*n* = 190).

Substance^a^	Bacterial species	No (%) of isolates with MIC (μg/mL)															
		≤ 0.0156	0.03125	0.0625	0.125	0.25	0.5	1	2	4	8	16	32	64	128	256	512
TCS	*Enterococcus* (*n* = 57)	1 (1.75)	2 (3.51)	1 (1.75)	3 (5.26)	3 (5.26)	1 (1.75)	3 (5.26)	2 (3.51)	4 (7.02)	**37** (64.91)	–	–	–	–	–	–
	*Staphylococcus* (*n* = 74)	**36** (48.65)	1 (1.35)	1 (1.35)	2 (2.7)	3 (4.05)	7 (9.46)	8 (10.81)	16 (21.62)	–	–	–	–	–	–	–	–
	*Klebsiella* (*n* = 2)	–	–	–	2 (100)	–	–	–	–	–	–	–	–	–	–	–	–
	*Acinetobacter* (*n* = 39)	**11** (28.21)	**8** (20.51)	6 (15.38)	5 (12.82)	–	3 (7.69)	2 (5.13)	1 (2.56)	3 (7.69)	–	–	–	–	–	–	–
	*Pseudomonas* (*n* = 10)	–	–	–	1 (10)	–	–	–	5 (50)	1 (10)	–	–	–	–	–	3 (30)	–
	*E.coli* (*n* = 8)	3 (37)	2 (25)	2 (25)	1 (12.5)	–	–	–	–	–	–	–	–	–	–	–	–
CHX	*Enterococcus* (*n* = 57)	–	–	–	–	–	–	1 (1.75)	**10** (17.54)	**18** (31.58)	**14** (24.56)	12 (21.05)	–	2 (3.51)	–	–	–
	*Staphylococcus* (*n* = 74)	–	–	–	1 (1.35)	–	–	19 (25.68)	**34** (45.95)	18 (24.32)	2 (2.7)	–	–	–	–	–	–
	*Klebsiella* (*n* = 2)	–	–	–	–	–	–	–	–	1 (50)	1 (50)	–	–	–	–	–	–
	*Acinetobacter* (*n* = 39)	–	–	–	–	–	–	1 (2.56)	2 (5.13)	8 (20.51)	5 (12.82)	9 (23.08)	6 (15.38)	7 (17.95)	1 (2.56)	–	–
	*Pseudomonas* (*n* = 10)	–	–	–	–	–	–	–	–	1 (10)	4 (40)	1 (10)	–	–	–	4 (40)	–
	*E.coli* (*n* = 8)	–	–	–	–	–	–	–	6 (75)	1 (12.5)	–	1 (12.5)	–	–	–	–	–
BKC	*Enterococcus* (*n* = 57)	–	–	–	–	–	–	–	6 (10.53)	**12** (21.05)	**16** (28.07)	**19** (33.33)	2 (3.51)	1 (1.75)	1 (1.75)	–	–
	*Staphylococcus* (*n* = 74)	–	–	–	–	–	–	4 (5.41)	**16** (21.62)	**15** (20.27)	**27** (36.49)	6 (8.11)	1 (1.35)	5 (6.76)	–	–	–
	*Klebsiella* (*n* = 2)	–	–	–	–	–	–	–	–	–	–	1 (50)	1 (50)	–	–	–	–
	*Acinetobacter* (*n* = 39)	–	–	–	–	–	–	–	2 (5.13)	2 (5.13)	5 (12.82)	**17** (43.59)	9 (23.08)	3 (7.69)	–	1 (2.56)	–
	*Pseudomonas* (*n* = 10)	–	–	–	–	–	–	–	–	–	–	3 (30)	2 (20)	–	1 (10)	1 (10)	3 (30)
	*E.coli* (*n* = 8)	–	–	–	–	–	–	–	–	–	–	–	5 (62.5)	2 (25)	1 (12.5)	–	–

^a^TCS, triclosan; CHX, chlorhexidine digluconate; BKC, benzalkonium chloride.

Bold number, the most frequent MIC for each disinfectant/bacterial species combination.

Most *Enterococcus* isolates exhibited CHX MICs ranging from 2 to 16 μg/mL (54/57, 94.7%), while *Staphylococcus* isolates showed pronounced concentration at 2 μg/mL (34/74, 45.95%). In contrast, CHX MICs for *Acinetobacter* spanned a broader range with most isolates distributed between 4 and 64 μg/mL.

BKC MICs of *Enterococcus* clustered at 4–16 μg/mL (47/57, 82.5%), whereas those for *Staphylococcus* clustered between 2 and 8 μg/mL (58/74, 78.4%). *Acinetobacter* displayed a wider range of BKC MICs (2–256 μg/mL), with a higher number at 16 μg/mL (17/39, 43.6%).

### 3.6 Association among susceptibilities of antibiotics and disinfectants

In *Enterococcus*, only CHX MICs were significantly associated with resistance to CHL, ERY, STR, TET and VAN (*p* < 0.05; Table 7). For *Staphylococcus*, MICs of all disinfectants were significantly associated with KAN resistance (*p* < 0.05). Additional significant associations were also observed for TCS and ERY, FUS, STR and TET; CHX and CIP and TET; and BKC and CLI, ERY and TIA (*p* < 0.05). In *Acinetobacter*, only TCS showed a significant association with CTX resistance (*p* < 0.05).

**Table 7:** Statistical association between disinfectant MICs and antibiotic resistance and correlation between disinfectant and antibiotic MICs among *Enterocochcus, Staphylococcus* and *Acinetobacter* (*n* = 170).

Substance	Bacterial species	Antimicrobial resistance/Correlation coefficients of MICs^a^																		
		FOX	CHL	CIP	CLI	COL	CTX	DOX	ERY	FUS	GEN	KAN	IMI	LVX	SMX	STR	TET	TIA	TMP	VAN
TCS	*Enterococcus* (*n* = 57)	nd	–/−0.03	nd	nd	nd	nd	nd	–/0.216	nd	nd	nd	nd	nd	nd	–/0.484^*^	–/0.390^*^	nd	nd	–/0.241
	*Staphylococcus* (*n* = 74)	-/0.223	–/−0.144	–/−0.021	–/0.76	nd	nd	nd	+/0.262^*^	+/−0.079	–/0.173	+/0.273^*^	nd	nd	–/0.236^*^	+/−1.06	+/0.331^*^	–/0.008	–/0.16	–/−0.127
	*Acinetobacter* (*n* = 39)	nd	nd	–/−0.74	nd	–/−0.418^*^	+/−0.08	–/−0.409^*^	nd	nd	–/−0.311	nd	–/−0.121	–/−0.105	nd	nd	nd	nd	nd	nd
CHX	*Enterococcus* (*n* = 57)	nd	+/0.317^*^	nd	nd	nd	nd	nd	+/0.244	nd	nd	nd	nd	nd	nd	+/0.457^*^	+/0.519^*^	nd	nd	+/0.435^*^
	*Staphylococcus* (*n* = 74)	-/0.132	–/0.048	+/0.338^*^	–/−0.009	nd	nd	nd	–/0.083	–/0.013	–/0.338^*^	+/0.443^*^	nd	nd	–/0.308^*^	– 0.232^*^	+/0.389^*^	–/−0.061	–/0.321^*^	–/0.099
	*Acinetobacter* (*n* = 39)	nd	nd	–/−0.182	nd	–/−0.144	–/0.14	–/0.268	nd	nd	–/0.226	nd	–/−0.331^*^	–/−0.102	nd	nd	nd	nd	nd	nd
BKC	*Enterococcus* (*n* = 57)	nd	–/0.162	nd	nd	nd	nd	nd	–/0.259	nd	nd	nd	nd	nd	nd	–/0.259	–/0.209	nd	nd	–/0.189
	*Staphylococcus* (*n* = 74)	-/0.132	–/0.132	–/0.338^*^	+/−0.009	nd	nd	nd	+/0.083	–/0.013	–/0.338^*^	+/0.443^*^	nd	nd	–/0.308^*^	–/0.232^*^	–/0.389^*^	+/−0.061	–/0.321^*^	–/0.099
	*Acinetobacter* (*n* = 39)	nd	nd	–/−0.451^*^	nd	–/0.155	–/0.067	–/0.001	nd	nd	–/−0.015	nd	–/−0.342^*^	–/−0.426^*^	nd	nd	nd	nd	nd	nd

+ Significant association between disinfectant MICs and antibiotic resistance (*p* < 0.05).

– No significant association between disinfectants MICs and antibiotic resistance (*p* > 0.05).

+ positive correlation (a value greater than 0) between MIC values of disinfectants and antibiotics that is the value of increase or decrease together, strong correlation means correlation coefficient close to 1.

– negative correlation (a value less than 0) between MIC values of disinfectants and antibiotics that is the one variable increase, the value of the other variable decreases, strong correlation means correlation coefficient close to −1.

^*^Significant correlation between MIC values of disinfectants and antibiotics (*p* < 0.05).

nd, not done.

^a^FOX, cefoxitin; CHL, chloramphenicol; CIP, ciprofloxacin; CLI, clindamycin; COL, colistin; CTX, cefotaxime; DOX, doxycycline; ERY, erythromycin; FUS, fusidate; GEN, gentamicin; IMI, imipenem; KAN, kanamycin; LVX, levofloxacin; SMX, sulfamethoxazole; STR, streptomycin; TET, tetracycline; TIA, tiamulin; TMP, trimethoprim; VAN, vancomycin; TCS, triclosan; CHX, chlorhexidine digluconate; BKC, benzalkonium chloride.

Correlations between MICs of disinfectants and antibiotics were further analyzed for *Enterococcus, Staphylococcus*, and *Acinetobacter* (Table 7). Among the statistically significant associations (*p* < 0.05), positive correlations were more frequently observed than negative correlations. Strong positive correlations (*r* > 0.5) were observed between MICs of TCS and GEN (*r* = 0.575), as well as between MICs of CHX and TET (*r* = 0.519) in *Enterococcus*. For *Staphylococcus*, positive correlations of low to moderate strength (*r* = 0.232–0.443) were observed, of which KAN MICs were positively correlated with those of TCS, CHX and BKC (Table 7). Interestingly, only negative correlations were observed for *Acinetobacter*.

### 3.7 Transferability of R plasmid and conjugation rate

According to the results of the conjugation experiments, AMP resistance was successfully transferred from donors *Acinetobacter* isolates: AC12.2, AC23.1, and AC43.2 to the recipient strain *E. coli* MG1655 rif^R^. The transfer occurred at a conjugation rate of 9.4 × 10^−9^ transconjugants per recipient cell. Similarly, COL resistance was horizontally transferred from donors *Acinetobacter* isolates AC18.1, AC59.1, and AC69.1 to the same recipient strain, also with a conjugation rate of 9.4 × 10^−9^. Transconjugants exhibited higher MIC values for the corresponding antibiotics than the donor strains (Table 8).

**Table 8:** MIC values of transconjugants (*n* = 6).

Donor^a^	MIC (μg/mL)		Selective pressure^b^	Transconjugant	MIC (μg/mL)	
	COL	AMP			COL	AMP
AC12.2	–	64	AMP	AC12.2_AMP	–	256
AC23.1	–	32	AMP	AC23.1_AMP	–	256
AC43.2	–	64	AMP	AC43.2_AMP	–	256
AC18.1	0.25	–	COL	AC18.1_COL	1	–
AC59.1	0.5	–	COL	AC59.1_COL	1	–
AC69.1	0.25	–	COL	AC69.1_COL	1	–

–, not done.

^a^AC, Acinetobacter spp.

^b^AMP, ampicillin; COL, colistin.

## 4 Discussion

The microbiological quality of indoor air in veterinary hospitals/clinics reflects complex clinical and environmental interactions, with important implications for infection control and occupational health at the human–animal interface. In this study, the majority of treatment rooms (90.3%) and inpatient wards (90.8%) exhibited total indoor microbial loads below the WHO-suggested limit, indicating adequate environmental hygiene and ventilation. A small proportion of treatment rooms (9.7%) and inpatient wards (9.2%) exhibited the high level airborne microbial loads. However, the specific drivers underlying elevated bacterial concentrations in these rooms were not investigated in this study and warrant further investigation, particularly factors related to animal density, movement patterns, ventilation performance, and cleaning practices.

In this work, airborne bacterial concentrations ranging from 33.37 to 2,881.82 CFU/m3 is in agreement with previous reports from veterinary teaching hospitals in Taiwan and a small-animal hospitals in Thailand (39, 40). At the same time, inpatient wards showed higher mean microbial concentrations (1,550.8 CFU/m3) compared with treatment rooms (1,243.8 CFU/m3), likely due to prolonged animal stays, frequent staff movement, and restricted ventilation efficiency, which collectively facilitate the accumulation of airborne bacteria. These observations aligned with previous studies in inpatient wards in human hospitals in Southern Ethiopia (41) and Southern Thailand (42). These results warrant particular attention, as airborne microbial contamination represents a potential route for exposure and transmission within clinical settings.

Lower relative humidity and temperature were associated with higher airborne bacterial load in this study, similar to previous studies (39, 43). Low relative humidity is known to enhance bacterial survival in aerosols by limiting hygroscopic growth and particle settling, thereby increasing airborne persistence (39). In contrast, the role of low temperature should be interpreted cautiously. Lower temperatures may contribute to increased airborne bacterial loads by slowing bacterial inactivation and reducing environmental stress, although their effects are species-dependent and less pronounced than those of relative humidity (43). Additionally, the narrow temperature range observed during sampling (27.5–30.1 °C) suggests that temperature likely had a limited influence in this study.

Gram-positive bacteria, especially *Staphylococcus* spp., were the most frequently isolated microorganisms consistent with previous studies in both human and veterinary healthcare settings (41, 44). This likely reflects their continuous shedding from animals and personnel and their role as common opportunistic pathogens in companion-animal practice, as well as their structural resilience to desiccation and oxidative stress (35, 42). It is worth noting that the findings were derived from enriched selective culture and therefore do not represent the full spectrum of bacterial populations recovered from air samples. As bacterial identification data was not obtained from non-selective cultures (45), it is likely that a substantial proportion of the recovered isolates were non-pathogenic bacteria. Despite the lower prevalence of Gram-negative bacteria in airborne dust, the detection of *Acinetobacter* spp. remains concerning because of their marked environmental persistence and association with hospital-acquired infections in both human and veterinary settings (46, 47).

The presence of airborne AMR bacteria represents a significant public health and animal health concern, particularly those exhibiting resistance to antimicrobials of critical importance in both human and veterinary medicine. Due to the limited availability of airborne AMR data from veterinary hospitals/clinics, comparative interpretation in this study relied on environmental surface studies in veterinary settings and airborne AMR investigations from human healthcare settings. Staphylococcal infection is among the most common clinical presentations in dogs and cats, the detection of resistant *Staphylococcus* spp. in airborne dust is of particular concern, as it represents a potential source of nosocomial exposure and compromises treatment efficacy (48). The observed resistance to β-lactams, tetracyclines, and macrolides in *Staphylococcus* is consistent with selection pressure associated with their frequent empirical use in small-animal practice, in particular for dermatologic and postoperative infections (49, 50). Similar observations were reported in an environmental surface study in veterinary hospitals/clinics and airborne dust from human hospitals (41, 51–53). Importantly, FOX-resistant *Staphylococcus* was detected. As FOX resistance is widely used as a phenotypic marker of methicillin resistance (MRSA or MRCoNS), such isolates are resistant to all β-lactam antibiotics, making their detection clinically and epidemiologically significant.

In spite of the limited findings of *Acinetobacter* isolates, their resistance to IMI and MEM is of particular concern, as Carbapenems are last-line antimicrobials reserved exclusively for the treatment of severe infections in humans and are not recommended for use in animals (54). This risk is further amplified by the ability of *Acinetobacter* spp. to persist in aerosols and on dry surfaces, facilitating maintenance and dissemination of clinically important resistance traits (55). The detection of TGC-resistant *Acinetobacter* is also noteworthy, with resistance significantly associated with inpatient wards (*p* < 0.05). TGC remains an important treatment option for MDR *Acinetobacter* infections, particularly when carbapenem resistance restricts therapeutic choices. Although not a first-line agent and limited by low serum concentrations, TGC is commonly used as a salvage or combination therapy for severe and complicated infections (56). The role of airborne *Acinetobacter* spp. in the dissemination of AMR within veterinary clinical environments was supported by their demonstrated ability to horizontally transfer AMP and COL resistance to *E. coli* in conjugation experiments.

COL-resistant *E. coli* was detected at low frequency in airborne dust from inpatient wards. This finding is of concern because COL is a last-resort antibiotic used to treat infections caused by MDR *E. coli*, particularly carbapenem-resistant strains. In addition, the potential for horizontal transfer of *mcr* genes poses a substantial risk for wider dissemination within and across bacterial species. However, COL has not been used in the participating veterinary hospitals (personal communication). This suggests that the observed resistance may be attributable to co-selection or cross-resistance driven by other antimicrobial pressures, dissemination from human or animal carriers, or persistence and circulation in the surrounding environment.

Vancomycin resistance was identified in a non-*E. faecium* isolate from an inpatient ward. This remains clinically and epidemiologically relevant, despite its generally lower impact compared with *E. faecium* (57). Its occurrence, particularly in immunocompromised hosts, warrants reporting and appropriate interpretation within AMR surveillance frameworks from a One Health perspective. However, the presence and transferability of *van* genes was not pursued in this study. The presence of bacterial pathogens resistant to clinically important antibiotics in airborne dust suggests a previously underrecognized pathway for environmental persistence and dissemination of AMR, with potential for inhalational exposure and indirect transmission between animals, humans, and the environment (58).

The presence of MDR airborne bacteria in indoor air of veterinary hospitals/clinics is of particular concern, as it demonstrates that resistant organisms are present not only on surfaces but also within the aerosolized clinical environment. This finding has important implications for healthcare environments, since airborne dust can facilitate the spread of MDR bacteria and increase inhalational exposure risks among veterinary personnel, animal handlers, and hospitalized animals (59). Moreover, the detection of airborne MDR bacteria, especially those resistant to critically important antimicrobials, may complicate infection control, increase the likelihood of treatment failure, and contribute to the wider environmental spread of AMR (60).

Overall, *Enterococcus, Staphylococcus* and *Acinetobacter* isolates exhibited minimal deviations in MIC distributions for all disinfectants TCS, CHX, and BKC with a single MIC value observed for certain compounds. Therefore, the isolates in this study appear to exhibit no or only limited reduced susceptibility to these disinfectants. The predominantly low TCS MICs observed for *Enterococcus, Acinetobacter* and *Staphylococcus* isolates are in agreement with previous studies in animal clinical isolates (61, 62) and may be attributable to triclosan's specific inhibition of FabI, which encodes the enoyl-acyl carrier protein (ACP) reductase involved in bacterial fatty acid biosynthesis at low concentrations (63). In contrast, *Pseudomonas* exhibited markedly elevated TCS MICs up to 256 μg/mL, consistent with its well-recognized intrinsic resistance mechanisms, including reduced outer membrane permeability and active efflux systems (64). These findings suggest limited efficacy of TCS against non-fermenting Gram-negative bacteria and raise concerns of its continued use in settings where such organisms are prevalent.

CHX and BKC showed narrower MIC distributions in *Enterococcus* and *Staphylococcus*, whereas broader and right-shifted distributions were observed in *Acinetobacter*. The wider MIC ranges for *Acinetobacter* may reflect the expression of efflux pumps and membrane-associated resistance mechanisms that confer reduced susceptibility to cationic disinfectants (65). In the context of airborne dust, these associations are of particular concern, as dust particles can act as reservoirs and vectors for disinfectant-tolerant and MDR bacterial species. Collectively, these findings indicate that disinfectant susceptibility is species-dependent, consistent with a previous study (66). The consistent correlations between disinfectant MICs and AMR suggest that routine disinfectant use in veterinary environments may contribute to increased antibiotic resistance in airborne bacteria, in line with a previous report (67).

Significant associations between disinfectant MICs and AMR further support the possibility of co-selection and cross-resistance (*p* < 0.05). In *Enterococcus*, such associations were limited to CHX that correlated with resistance to multiple clinically important antimicrobials (i.e., CHL, STR, VAN, and TET), consistent with a previous study indicating that CHX exposure can alter cell membrane permeability and induce multidrug efflux systems, thereby reducing intracellular accumulation of diverse antibiotic classes (68). In contrast, *Staphylococcus* exhibited broader associations, most notably between KAN resistance and MICs of all tested disinfectants. Together with additional associations involving macrolides, tetracycline, and fluoroquinolones, this pattern suggests that disinfectant exposure may select for strains carrying mobile genetic elements or multidrug efflux systems that mediate cross-resistance (62). Additionally, *Acinetobacter* demonstrated a significant association between TCS MIC and CTX resistance. This may not be surprising because *A. baumannii* exhibits intrinsic or acquired resistance to third-generation cephalosporins due to β-lactamase production and reduced outer-membrane permeability (69).

In this study, limitations include the lack of antibiotic use history and detailed information on ventilation management, as well as routine cleaning and disinfection practices, which could have facilitated a more robust interpretation of the results. Air sampling was conducted on a single occasion at each facility, and key variables that may influence airborne bacterial load such as time of day, human and animal activity levels, and environmental conditions were not standardized. These methodological constraints may have introduced variability in bacterial recovery and limit the representativeness and generalizability of the findings to overall environmental contamination. Furthermore, the absence of standardized interpretive criteria for disinfectant susceptibility limits the ability to define resistance thresholds and to directly compare findings across studies.

In conclusion, indoor airborne dust in veterinary facilities may act as a reservoir for bacterial pathogens and AMR, posing potential risks to both animal and human health. Disinfectant use potentially contribute to shaping AMR profiles in airborne dust-associated bacteria, emphasizing that selective pressures in the built environment extend beyond antibiotic use alone. Strengthening antimicrobial stewardship in small-animal practice, together with improved infection control and ventilation strategies, is therefore essential to mitigate airborne dissemination, nosocomial exposure risks and the propagation of resistance within the One Health framework. These findings support the implementation of baseline bioaerosol sampling to establish background levels of airborne contamination in veterinary facilities. Routine AMR bioaerosol monitoring in veterinary hospitals may be justified under specific circumstances for example, recurrent outbreaks, high-risk areas, management of severe infections, or concerns about airborne dissemination. A risk-based approach comprising baseline assessment, targeted sampling during outbreaks or when unusual resistance patterns are detected, and integrating the findings into existing infection prevention and AMR surveillance is more practical and cost-effective. Incorporating disinfectant susceptibility testing could further strengthen comprehensive AMR data generation.

## Data Availability

The original contributions presented in the study are included in the article, further inquiries can be directed to the corresponding author.
